# Substance use and HIV stage at entry into care among people with HIV

**DOI:** 10.1186/s13690-021-00677-2

**Published:** 2021-08-28

**Authors:** Canada Parrish, Bridget M. Whitney, Robin M. Nance, Nancy Puttkammer, Paul Fishman, Katerina Christopoulos, Julia Fleming, Sonya Heath, William Christopher Mathews, Geetanjali Chander, Richard D. Moore, Sonia Napravnik, Allison Webel, Joseph Delaney, Heidi M. Crane, Mari M. Kitahata

**Affiliations:** 1 University of Washington, 1705 NE Pacific Street, Seattle, WA 98195 USA; 2 University of California San Francisco, San Francisco, CA USA; 3 Fenway Institute, Boston, MA USA; 4 University of Alabama at Birmingham, Birmingham, AL USA; 5 University of California San Diego, San Diego, CA USA; 6 Johns Hopkins University, Baltimore, MD USA; 7 University of North Carolina at Chapel Hill, Chapel Hill, NC USA; 8 Case Western Reserve University, Cleveland, OH USA; 9 University of Manitoba, Winnipeg, MB Canada

**Keywords:** Substance use, Early entry into care, Risk factors

## Abstract

**Abstract:**

**Background:**

Information regarding the impact of substance use on the timing of entry into HIV care is lacking. Better understanding of this relationship can help guide approaches and policies to improve HIV testing and linkage.

**Methods:**

We examined the effect of specific substances on stage of HIV disease at entry into care in over 5000 persons with HIV (PWH) newly enrolling in care. Substance use was obtained from the AUDIT-C and ASSIST instruments. We examined the association between early entry into care and substance use (high-risk alcohol, methamphetamine, cocaine/crack, illicit opioids, marijuana) using logistic and relative risk regression models adjusting for demographic factors, mental health symptoms and diagnoses, and clinical site.

**Results:**

We found that current methamphetamine use, past and current cocaine and marijuana use was associated with earlier entry into care compared with individuals who reported no use of these substances.

**Conclusion:**

Early entry into care among those with substance use suggests that HIV testing may be differentially offered to people with known HIV risk factors, and that individuals with substances use disorders may be more likely to be tested and linked to care due to increased interactions with the healthcare system.

## Background

In the era of universal treatment for HIV, increasing attention has been focused on the HIV continuum of care from diagnosis to linkage and retention in care, initiation of antiretroviral therapy (ART), and achievement of HIV viral suppression [1, 2]. Timely diagnosis and engagement in care are key to maximizing the benefit of ART, minimizing the long-term negative consequences of HIV, and the success of a treatment as prevention approach [3–5]. In the United States (US), it is estimated that only 86% of people with HIV (PWH) have been diagnosed, and only 64% have been linked to care [6]. Although these proportions change over time, they are well below national and international HIV targets [2].

Traditional care models may pose challenges for PWH affected by factors that hinder the ability to engage in care on a routine basis [7–9]. Drug and alcohol use disorders often prevent PWH from reliably seeking follow-up care after HIV diagnosis or during the lifelong HIV treatment process [10–13]. In some regions, PWH who inject drugs represent a substantial portion of the HIV-infected population, which may explain ongoing HIV transmission and limited progress towards global HIV targets [2, 14, 15]. Research is needed to better define the challenges and optimal approaches to improve care delivery to individuals with co-occurring substance use.

Factors that have been associated with delayed HIV testing and linkage to care include younger age, Black or Hispanic race/ethnicity, lower socioeconomic status, and HIV testing without co-located HIV care services [3, 5, 16, 17]. However, these studies are somewhat dated as testing policies have continued to evolve, and the role of substance use has been less extensively examined [10, 18]. Little is known about the impact of specific substances or combinations of substances on the timing of linkage to care. Information regarding the relationship between substance use by substance type and the early stages of the HIV care continuum is limited. In this study, we used comprehensive clinical data from a geographically diverse cohort of PWH to characterize the effect of specific substances and multi-substance use on the timing of entry into HIV care.

We conducted this study to identify factors, particularly the impact of substance use, associated with differential engagement in early steps of the HIV care cascade among PWH in real-world settings. By examining when in their course of HIV disease PWH initiate care, we can better understand the impact of factors such as substance use on the initial HIV care cascade steps of testing and linkage. Additionally, care policies no longer target testing to high-risk groups but instead encourage HIV testing for everyone [19]. A better understanding of factors associated with entry into care, can help guide approaches and policies to improve HIV testing and linkage.

We examined the association between entry into care at earlier or later stages of HIV disease, defined by higher vs. lower CD4 counts (≥350 cells/mm^3^ compared to < 350 cells/mm^3^), and substance use and/or high-risk alcohol use. Substance use refers to use of illegal drugs, drugs taken for reasons or at amounts other than prescribed (e.g. illicit opioid use), drugs that may be federally banned, but permitted by certain states (marijuana) and alcohol. We further defined alcohol use here as “high-risk” alcohol use [20], as moderate or occasional alcohol consumption would not be expected to influence care seeking behaviors. We hypothesized that PWH reporting current substance use would be less likely to initiate care early (at higher CD4 counts) than PWH who do not use substances or high-risk alcohol. We further hypothesized that this relationship would vary depending on substance type and frequency of use, as well as different between sociodemographic sub-groups.

## Methods

### Data source

The Centers for AIDS Research (CFAR) Network of Integrated Clinical Systems (CNICS) cohort is a longitudinal observational study of adult PWH receiving care at eight clinical sites across the US from 1995 to the present [18, 21]. The CNICS cohort is geographically diverse with demographic and clinical characteristics similar to the overall population of PWH in the US [6]. Comprehensive clinical data collected through electronic data systems undergo rigorous quality assessment, are harmonized in a central repository, and are updated on a quarterly basis [21]. These data include demographic information, laboratory data, antiretroviral medication data, and diagnosis data including diabetes, hypertension, cardiovascular disease, mental illness, and substance use. Additionally, symptoms and behaviors are self-reported longitudinally by the CNICS clinical assessment of patient-reported measures and outcomes (PROs) using validated instruments including the PHQ-9 for depression [22], the AUDIT-C (Alcohol Use Disorders Identification Test) for alcohol use [23], and the ASSIST (modified Alcohol, Smoking, and Substance Involvement Screening Test) for substance use [24]. The CNICS clinical assessment of PROs are administered approximately every 6 months [25] via tablets as part of routine clinical care. CNICS data adheres to HIPAA, and was anonymized for the current study for the protection of the privacy of the subjects.

### Study population

We examined all PWH newly enrolling in HIV care from January 1, 2010 to September 30, 2019. We included PWH enrolled in CNICS in or after 2010 in order to reflect current treatment and clinical care practices. PWH with evidence of previous HIV care prior to CNICS entry (e.g. historical ART or undetectable viral loads) were excluded. The analytic sample included PWH who completed the CNICS clinical assessment of PROs within the first 12 months of entry into care. Initiation of the clinical assessment varied across sites/institutions (site *N* = 8), so the study period start date also varied by site (median year of initiation 2011).

### Statistical analysis

#### Study design and primary outcome

We conducted a cross-sectional analysis of HIV disease stage measured by CD4 count at the time of care initiation as the primary outcome of interest. Early entry into care was defined as starting clinical care for HIV with higher CD4 cell counts ≥350 cells/mm^3^ compared to later entry into care defined by lower CD4 cell counts < 350; CD4 cut points were informed by previous research and clinical relevance [10, 26–29].

#### Primary predictor or exposure

Substance use, the primary predictor of interest, was obtained from PROs using the AUDIT-C [20, 23] and ASSIST [24] instruments. The first AUDIT-C and ASSIST completed within the first year of care was used as a proxy of substance and alcohol use at the time of entry into care. High-risk alcohol use was defined by AUDIT-C scores of greater than or equal to 4 for men and 3 for women [20]. Methamphetamine, cocaine/crack, illicit opioid, and marijuana use were each categorized into three mutually exclusive categories: never used, past use, and current use. Illicit opioids includes all opioids that are prohibited by law or the use of prescription opioids not taken as prescribed.

#### Adjustment variables or covariates in the inferential analysis

Covariates of interest, including sex (assigned male or female at birth), age, race/ethnicity, mental health diagnoses, current depressive symptoms (measured by the PHQ-9), and CNICS site, were included in all statistical models as adjustment variables. There were a very small number of individuals recorded as transgender who were not included in the analysis due to sample size and protection of identify. For race/ethnicity, we constructed a four-category variable of: non-Hispanic White, non-Hispanic Black, Hispanic (of any race), and Other (any race, other than White or Black, who did not identify as Hispanic). For mental health diagnoses, we categorized PWH into one of three hierarchical groups [30]: 1) any psychotic disorder, bipolar disorder, and/or personality disorders with or without depression and/or anxiety, 2) depression and/or anxiety only, 3) no history of mental illness as documented within the first 6 months of entry into care.

The HIV acquisition risk factor was obtained directly from clinical records and noted as one of three categories: men who have sex with men (MSM), injection drug use (IDU), and heterosexual transmission. IDU was not included in the models due to collinearity with substances and difficulty of interpretation of substance coefficients in the presence of this variable. MSM was included as an HIV risk factor in the multiple substance model so that we could characterize the relationships of interest among two distinct male populations (MSM and non-MSM men) as substance use and HIV care seeking behaviors differ between these populations [31–33].

Additional variables included in sensitivity analyses were: year of entry into CNICS, enrollment in substance use treatment programs, hepatitis C virus coinfection, and comorbidities such as AIDS defining illnesses, diabetes, and hypertension. Year of entry was determined by the calendar year at which an individual first enrolled into the CNICS cohort. Enrollment in substance use treatment programs was a binary variable from responses in the PROs that signified if the individual had ever sought formal treatment of any substance use disorder. Hepatitis C and other comorbidities were indicators derived from diagnosis codes and/or condition specific medications for treatment as listed in the medical records.

#### Descriptive analysis

We compared the prevalence of use for each of the substances of interest at early or later initiation of care using chi square tests for categorical variables and t-tests for continuous variables.

#### Inferential analysis

We employed logistic regression models and relative risk regression models to determine factors associated with early entry into care compared to later entry into care; relative risk estimates from a generalized linear model with Poisson family and log link [34] were compared with the odds ratios obtained in the logistic regression models to aid in interpretability of associations since the outcome was common [35]. Only those with complete data were included in the regression models. All models used robust standard errors. Analyses were completed in Stata 14 (StataCorp 2015).

#### Inferential analysis: single substance models

Categorical parametrizations of substance use (never used, past use, current use) for methamphetamine, cocaine/crack, illicit opioid, and marijuana, and a binary parameterization of high-risk alcohol use (high-risk drinking vs. no high-risk drinking) were used to construct five separate, single-substance models. These models were designed to estimate the effect of *each* drug of interest and high-risk alcohol use separately. Additional analytic models included two-way multiplicative interaction terms for each substance of interest with age, sex, and race/ethnicity.

To address the impact of substance use frequency on the outcome, we included a second set of single substance models with measures of substance use frequency. These models included continuous variables for days of use in the last 30 days with adjustment for use of other substances (binary indicators of never/ever use for each substance).

#### Inferential analysis: multiple substance model

Finally, an analytic model including all reported substances and high-risk alcohol use was evaluated to assess the impact of *concurrent* use of substances on entry into care. In addition to adjusting for other current substance use, a multiple substance use indicator variable was included for any combination of substances or high-risk alcohol (not for specific combinations). Previous studies have suggested that multiple substance use is common among PWH [18]. An indicator for men who have sex with men (MSM) was also included in this model.

#### Sensitivity analyses

Sensitivity analyses were conducted with: 1.) other CD4 cut points (e.g. 500 cells/mm^3^), 2.) adjustment for other factors of interest that may potentially impact the relationship under examination such as year of entry into CNICS, enrollment in substance use treatment programs, hepatitis C virus coinfection, and other comorbidities (e.g. AIDS defining illnesses, diabetes, hypertension, etc.), 3.) linear regression models with continuous CD4 as the outcome, and 4.) models that varied the PRO window of eligibility. We also conducted a stratified analysis by HIV risk factors (i.e. MSM, IDU, heterosexual sex, or other/unknown; MSM who were also IDU were categorized into the MSM risk factor group) to assess the relationship between substance use and early entry into care for these specific populations.

## Results

Among 5017 PWH who entered into care from January 2010 through September 2019, the majority were male (86%) with a mean age of 40 years (standard deviation (SD): 12 years). Mental health diagnoses and past or current substance use were more prevalent than reported within the general US population [36]. As shown in Table 1, demographic characteristics and substance use varied by early or later entry into care in unadjusted analyses. Less than 1% of the sample had any missing data for the sociodemographic variables; missingness for the primary predictors of substances and high-risk alcohol use ranged from 1.6% for high-risk alcohol use to 7.8% missingness for illicit opioids. There was complete ascertainment of the outcome variable.

**Table 1:** Demographic and clinical characteristics including substance use of PWH in this study categorized by CD4 count at enrollment in care

PWH Characteristics	Overall (***N*** = 5017)	HIV Severity		
		CD4 < 350 cells/mm^**3**^ (***N*** = 1945)	CD4 ≥ 350 cells/mm^**3**^ (***N*** = 3072)	***P***-value^**a**^
**Age in years, mean (SD)**	40.2 (11.9)	40.9 (11.7)	39.2 (12.1)	< 0.01
**Male, N (%)**	4333 (86.4)	1669 (85.8)	2664 (86.8)	0.35
**Race/Ethnicity, N (%)**				< 0.01
Non-Hispanic White	2149 (42.8)	378 (37.9)	1411 (45.9)	
Non-Hispanic Black	1670 (33.3)	748 (38.5)	922 (30.0)	
Hispanic	850 (16.9)	336 (17.3)	514 (16.7)	
Other	348 (6.9)	123 (6.3)	225 (7.3)	
**Mental Health Diagnoses, N (%)**				< 0.01
No mental health diagnoses	2747 (54.8)	1153 (59.3)	1594 (51.9)	
Depression and/or anxiety only	1801 (35.9)	634 (32.6)	1167 (38.0)	
Psychosis, bipolar, personality disorders (with or without depression and/or anxiety)	469 (9.4)	158 (8.1)	311 (10.1)	
**Methamphetamines/crystal**				< 0.01
Never used	3136 (64.6)	1299 (69.4)	1837 (61.6)	
Past use	1142 (23.5)	140 (21.9)	732 (24.6)	
Current use	576 (11.9)	164 (8.8)	412 (13.8)	
**Cocaine/Crack**				< 0.01
Never used	2678 (54.7)	1123 (59.3)	1555 (51.8)	
Past use	1809 (36.9)	639 (33.7)	1170 (39.0)	
Current use	410 (8.4)	133 (7.0)	277 (9.2)	
**Marijuana**				< 0.01
Never used	1461 (29.8)	645 (33.8)	816 (27.2)	
Past use	1666 (34.0)	637 (33.4)	1029 (34.4)	
Current use	1775 (36.2)	624 (32.7)	1151 (38.4)	
**Illicit Opioids**				0.02
Never used	3832 (82.8)	1477 (84.8)	2355 (81.7)	
Past use	645 (13.9)	217 (12.5)	428 (14.8)	
Current use	149 (3.2)	48 (2.8)	101 (3.5)	
**High-risk Alcohol Consumption**				0.01
High-risk drinking	3859 (78.1)	1530 (80.0)	2329 (77.0)	
No high-risk drinking	1080 (21.9)	383 (20.0)	697 (23.0)	
**HIV Risk Factor**				< 0.01
Men who have Sex with Men (MSM)	3222 (64.2)	1182 (60.8)	2040 (66.4)	
Injection Drug Use (IDU)	557 (11.1)	207 (10.6)	350 (11.4)	
Heterosexual	1008 (20.1)	465 (23.9)	543 (17.7)	
Unknown or Other	230 (4.6)	91 (4.7)	139 (4.5)	

^a^Comparing PWH populations with CD4 < 350 and CD4 ≥ 350 cells/mm^3^

In the single substance models, we found a significant association between current methamphetamine use (OR = 1.52, 95% Confidence Interval (CI): 1.23–1.88; RR = 1.14, 95% CI: 1.07–1.22), as well as past and current cocaine and past and current marijuana use (ORs = 1.20, 1.43, 1.19, 1.33, respectively; RRs = 1.07, 1.13, 1.07, 1.12) and increased likelihood of early entry into care (CD4 count > 350 cells/mm^3^) compared with PWH who reported no use of those substances. Associations between early initiation into care and past methamphetamine use, past and current illicit opioid use, and high-risk alcohol consumption were not statistically significant (Table 2).

**Table 2:** Associations between substance use and early entry into care^a^ (individual models for each substance) in adjusted analyses^b^

Substance	OR	P-value	95% CI	RR	P-value	95% CI
**Methamphetamines (ref. Never used)**						
Past use only	1.09	0.29	0.93–1.27	1.03	0.28	0.98–1.09
*Current use*	**1.52**	**< 0.01**	**1.23–1.88**	**1.14**	**< 0.01**	**1.07–1.22**
**Cocaine/Crack (ref. Never used)**						
*Past use only*	**1.20**	**0.01**	**1.05–1.37**	**1.07**	**0.01**	**1.02–1.12**
*Current use*	**1.43**	**< 0.01**	**1.14–1.79**	**1.13**	**< 0.01**	**1.05–1.22**
**Marijuana (ref. Never used)**						
*Past use only*	**1.19**	**0.02**	**1.03–1.38**	**1.07**	**0.02**	**1.01–1.14**
*Current use*	**1.33**	**< 0.01**	**1.14–1.55**	**1.12**	**< 0.01**	**1.05–1.19**
**Illicit Opioids (ref. Never used)**						
Past use only	1.12	0.22	0.93–1.34	1.03	0.22	0.98–1.10
Current use	1.22	0.28	0.85–1.74	1.07	0.25	0.95–1.20
**High-risk Alcohol Consumption (ref. No high-risk drinking)**						
High-risk drinking	1.12	0.11	0.97–1.30	1.04	0.12	0.99–1.10

^a^ Early entry into care define as entering care with CD4 counts > 350 cells/mm^3^

^b^ All models adjusted for sex, age, race/ethnicity, current depressive symptoms, mental health diagnoses, and treatment site

None of the two-way interaction terms for each substance with age, sex, and race/ethnicity were statistically significant (data not shown), demonstrating no observed effect modification for any substance by demographic characteristics. In single substance models including measures of substance use frequency within the last 30 days, we found no statistically significant effects of greater frequency of substance use on early entry into care compared to lower frequency of use. There was not a strong dose response relationship between substance use and early care entry for any of the substances (Table 3).

**Table 3:** Single-substance models with frequency of use within the past 30 days and association with early entry into care^a,b^

Substance^**c**^	OR	***P***-value	95% CI	RR	P-value	95% CI
**Methamphetamines**	1.10	0.24	0.94–1.30	1.03	0.22	0.98–1.07
**Cocaine/Crack**	0.87	0.31	0.67–1.14	0.96	0.36	0.87–1.05
**Marijuana**	0.94	0.12	0.87–1.02	0.98	0.11	0.95–1.00
**Illicit Opioids**	0.80	0.16	0.58–1.10	0.93	0.19	0.83–1.04
**Alcohol**	1.00	0.80	0.97–1.02	1.00	0.82	0.99–1.00

^a^ Early entry into care define as entering care with CD4 counts > 350 cells/mm^3^

^b^ All models adjusted for sex, age, race/ethnicity, current depressive symptoms, mental health diagnoses, treatment site, and use of other substances

^c^ Substances are measured by days of use within the past month (range 0–30)

In the model that included all substances, the relationships between early entry into care and current methamphetamine, cocaine/crack, and marijuana use remained statistically significant and the magnitude of the associations did not change. After accounting for use of other substances, current methamphetamine (OR = 1.52, 95% CI: 1.16–1.98; RR = 1.14, 95% CI: 1.05–1.23), cocaine/crack (OR = 1.44, 95% CI: 1.07–1.93; RR = 1.12, 95% CI: 1.02–1.23), and marijuana (OR = 1.27, 95%CI: 1.04–1.53; RR = 1.09, 95% CI: 1.02–1.18) use were associated with earlier entry into care compared to PWH who did not use these substances in adjusted analyses. Past use of any of the substances was no longer associated with timing of care entry when controlling for use of the other substances and high-risk alcohol use (Table 4).

**Table 4:** Associations between substance use and early entry into care^a^ in adjusted analyses^b,c^

Substance	OR	P-value	95% CI	RR	P-value	95% CI
**Methamphetamines (ref. Never used)**						
Past use only	0.96	0.67	0.79–1.16	0.99	0.69	0.92–1.05
Current use	**1.52**	**0.00**	**1.16–1.98**	**1.14**	**< 0.01**	**1.05–1.23**
**Cocaine (ref. Never used)**						
Past use only	1.15	0.10	0.97–1.37	1.05	0.10	0.92–1.12
Current use	**1.44**	**0.02**	**1.07–1.93**	**1.12**	**0.01**	**1.02–1.23**
**Marijuana (ref. Never used)**						
Past use only	1.10	0.27	0.92–1.30	1.04	0.23	0.98–1.11
Current use	**1.27**	**0.02**	**1.04–1.53**	**1.09**	**0.01**	**1.02–1.18**
**Illicit Opioids (ref. Never used)**						
Past use only	1.04	0.73	0.85–1.27	1.01	0.79	0.94–1.08
Current use	1.02	0.94	0.68–1.53	1.00	0.95	0.89–1.14
**High-risk Alcohol Consumption (ref. No high-risk drinking)**						
High-risk drinking	1.17	0.12	0.96–1.42	1.05	0.12	0.99–1.23

^a^ Early entry into care define as entering care with CD4 counts > 350 cells/mm^3^

^b^All substances included in the same analytic model

^c^ All models adjusted for sex, age, race/ethnicity, MSM, current depressive symptoms, mental health diagnoses, and treatment site

In the full model, we found a significant association between older age, being male with no reported sex with men, Black race, and Hispanic ethnicity and lower likelihood of early entry into care. Men who have sex with men were more likely to enter care with higher CD4 counts than men who did not report same-sex sexual relationships. Those with mental health diagnoses were more likely to enter care early than those without mental health diagnoses. Current depressive symptoms were not independently associated with early care initiation when controlling for the other covariates. (Table 5).

**Table 5:** Associations^a^ between PWH Characteristics and Early Entry into Care^b^

Substance	OR	P-value	95% CI	RR	P-value	95% CI
**Male, Not MSM (ref. Female)**	**0.68**	**< 0.01**	**0.54–0.85**	**0.86**	**0.01**	**0.79–0.94**
**MSM**	**1.33**	**< 0.01**	**1.14–1.56**	**1.12**	**< 0.01**	**1.05–1.19**
**Age**	**0.99**	**0.01**	**0.98–0.99**	**0.99**	**0.01**	**0.99–0.99**
**Race/ethnicity (ref. Non-Hispanic White)**						
Non-Hispanic Black	**0.80**	**0.01**	**0.67–0.94**	**0.92**	**0.01**	**0.86–0.98**
Hispanic	**0.79**	**0.01**	**0.66–0.95**	**0.92**	**0.02**	**0.86–0.98**
**Current depressive symptoms (PHQ-9 Score)**	0.96	0.11	0.90–1.01	0.98	0.12	0.97–1.0
**Mental Health Diagnosis (ref. No Mental Health Diagnosis)**						
Depression and/or Anxiety	**1.26**	**< 0.01**	**1.09–1.46**	**1.09**	**< 0.01**	**1.03–1.15**
Any psychotic, bipolar, or personality disorder	**1.42**	**0.01**	**1.10–1.82**	**1.13**	**< 0.01**	**1.04–1.22**

^a^ Adjusted for all substances and treatment site

^b^ Early entry into care define as entering care with CD4 counts > 350 cells/mm^3^

Sensitivity analyses using different specifications of the outcome variable and inclusion of additional comorbidity and adjustment covariates yielded similar results to the models described above (data not shown). Variation of the time window in which a PRO assessment was attributed to baseline substance use and alcohol consumption also did not affect the results. Patterns in the associations between substance use and early entry into care were also similar in analyses stratified by HIV risk factor (MSM, IDU, heterosexual sex).

## Discussion

Contrary to our hypotheses, we found that PWH who reported current methamphetamine, cocaine/crack, or marijuana use were more likely to enter care at an earlier stage of HIV disease (as noted by CD4 counts ≥350 cells/mm^3^) than those who did not report use of these substances. However, the magnitude of the observed effects was modest. We did not find an association between illicit opioid or high-risk alcohol use and stage of disease at entry into care. Reported associations between early entry into care and substance or high-risk alcohol use did not differ by demographic characteristics, including age, sex, and race/ethnicity, nor by HIV risk factor.

A possible explanation for our findings is that HIV testing continues to be targeted to persons at higher risk of HIV infection, such as those with certain substance use disorders [31, 37], and that recommendations for universal testing for HIV have not been fully adopted in clinical practice [19]. People who report substance use may be more likely to be tested for HIV than those who do not report these behaviors [31, 38, 39], and more frequent testing can lead to earlier diagnosis of HIV and entry into care. This trend towards selective testing could be driven in part by self-selection by individuals with known HIV risk factors as education regarding high-risk behaviors can increase motivation for testing [31, 37, 40], and by clinicians who may be reluctant to routinely test all patients for HIV due to lack of awareness of newer testing guidelines or perceived limited capacity to integrate routine testing into practice [39].

In addition, substance use may increase engagement with the health care system due to exacerbation of underlying health conditions, overdose, injuries related to impairment [41–43], and/or substance use treatment, that provide more opportunities for testing and care initiation. Previous studies have found an association between physical health comorbidities and substance use [41, 43], which may prompt greater interaction with the health care system. While we controlled for hypertension, diabetes, and hepatitis C coinfection in sensitivity analyses, there may be additional health conditions that influence early entry into care in our study that we did not take into account. Additionally, clinical outreach programs targeting people who are homeless may bring certain subsets of people into clinical settings at earlier HIV disease stages, and the relationship between homelessness and substance use is well-established [44].

There are limitations of this study. We were interested in examining factors associated with entry into care and therefore excluded PWH with documented evidence of prior treatment. However, there is the possibility of misclassification if evidence of prior treatment was not well-documented. Second, detailed substance use information was obtained from the CNICS clinical assessment of PROs, and not all PWH have PRO assessments within the first year of care. This could result in potential for selection bias if those with substance use disorders and high-risk alcohol use and lower CD4 counts were less likely to receive PRO assessments systematically across CNICS clinics. However, sensitivity analyses using varying time windows for PRO completion, including 18 and 24 months, found similar results, reducing our concern about selection bias. Additionally, PROs are only available in English, Spanish, and Amharic, so not all PWH may have been able to complete a PRO in their preferred language. Third, the timing of starting PRO assessment at sites varied so not all sites contributed participants throughout the entire study period. Fourth, the prevalence of illicit opioid use in the CNICS cohort is low, so non-significant results between illicit opioid use and stage of entry into care may be, in part, due to statistical power issues. Finally, findings may not generalize to other treatment contexts as a large proportion of PWH in the CNICS cohort live in urban and suburban settings.

This research has notable strengths too, including detailed substance use information using validated instruments collecting current and past substance use; demographic, clinical, and geographic diversity; and data reflecting clinical practice in the current treatment era.

## Conclusions

This study provides insight as to the impact of specific substance use, as well as high-risk alcohol, on the HIV stage at which a person enters care for HIV. Early entry into care among PWH who use substances suggests that HIV testing may still be differentially offered to people with known HIV risk factors in routine care and in targeted outreach, and that individuals with substances use disorders may be more likely to be tested and linked to care due to increased interactions with the healthcare system. It is likely that a combination of factors is driving the associations we observed. Our findings also indicate that there is room for improvement in testing and linkage to care overall and in particular among those without documented risk factors for HIV.

## Data Availability

The data analyzed during the current study are not publicly available due HIPPA protections of medical records data.

## Abbreviations

ART: Antiretroviral therapy
PHW: Persons with HIV
CNICS: The Centers for AIDS Research (CFAR) Network of Integrated Clinical Systems
PROs: Patient Reported Outcomes
AUDIT-C: Alcohol Use Disorders Identification Test
ASSIST: Alcohol, Smoking, and Substance Involvement Screening Test
IDU: Injection Drug Use
MSM: Mem who have Sex with Men

## References

[1] Sidibé M, Loures L, Samb B (2016). The unaids 90-90-90 target: a clear choice for ending aids and for sustainable health and development. J Int AIDS Soc.

[2] Levi J, Raymond A, Pozniak A, Vernazza P, Kohler P, Hill A. Can the UNAIDS 90–90-90 target be achieved? A systematic analysis of national HIV treatment cascades. BMJ Glob Heal. 2016;1(2):e000010. Available from: http://gh.bmj.com/lookup/doi/10.1136/bmjgh-2015-000010 10.1136/bmjgh-2015-000010 PMC5321333 28588933

[3] Ulett KB, Willig JH, Lin H, Ph D, Routman JS, Abroms S, et al. The Therapeutic Implications of Timely Linkage and Early Retention in HIV Care. AIDS Patient Care STDS. 2009;23(1):41-49. 10.1089/apc.2008.0132 PMC2733237 19055408

[4] Moore RD. Epidemiology of HIV Infection in the United States : Implications for Linkage to Care. 2011;52(Suppl 2). 10.1093/cid/ciq044 PMC3106255 21342909

[5] Maartens G, Celum C, Lewin SR. HIV infection: Epidemiology, pathogenesis, treatment, and prevention. In: The Lancet. Elsevier Ltd; 2014. p. 258–271. Available from: 10.1016/S0140-6736(14)60164-1 10.1016/S0140-6736(14)60164-1 24907868

[6] CDC (2019). Monitoring selected national HIV prevention and care objectives by using HIV surveillance data. HIV Surveill Suppl Rep.

[7] Holmes CB, Sanne I (2015). Changing models of care to improve progression through the HIV treatment cascade in different populations. Curr Opin HIV AIDS.

[8] Bonevski B, Randell M, Paul C, Chapman K, Twyman L, Bryant J, et al. Reaching the hard-to-reach: A systematic review of strategies for improving health and medical research with socially disadvantaged groups. BMC Med Res Methodol. 2014;14(1):1–29. 10.1186/1471-2288-14-42 PMC3974746 24669751

[9] Gonsalves GS, Paltiel AD, Cleary PD, Gill MJ, Kitahata MM, Rebeiro PF (2017). A flow-based model of the HIV care continuum in the United States. J Acquir Immune Defic Syndr.

[10] Palepu A, Horton N, Tibbetts N, Meli S, Samet J (2004). Uptake and adherence to highly active antiretroviral therapy among HIV-infected people with alcohol and other substance use problems: the impact of substance abuse treatment. Addiction.

[11] Crane HM, McCaul ME, Chander G, Hutton H, Nance RM, Delaney JAC (2017). Prevalence and factors associated with hazardous alcohol use among persons living with HIV across the US in the current era of antiretroviral treatment. AIDS Behav.

[12] Monroe AK, Lau B, Mugavero MJ, Mathews WC, Mayer KH, Napravnik S (2016). Heavy alcohol use is associated with worse retention in HIV care. J Acquir Immune Defic Syndr.

[13] Mugavero MJ, Westfall AO, Cole SR, Geng EH, Crane HM, Kitahata MM (2014). Beyond core indicators of retention in HIV care: missed clinic visits are independently associated with all-cause mortality. Clin Infect Dis.

[14] Des Jarlais DC, Kerrr T, Carrieri P, Feelemyer J, Arasteh K. HIV Infection among Persons who inject Drugs: Ending Old Epidemics and Addressing New Outbreakscess 2017;30(6):815–826. 10.1097/QAD.0000000000001039 PMC4785082 26836787

[15] May MT, Justice AC, Birnie K, Ingle SM, Smit C, Smith C (2015). Injection drug use and hepatitis C as risk factors for mortality in HIV-infected individuals: the antiretroviral therapy cohort collaboration. J Acquir Immune Defic Syndr.

[16] Govindasamy D, Meghij J, Negussi EK, Baggaley RC, Ford N, Kranzer K. Interventions to improve or facilitate linkage to or retention in pre-ART (HIV) care and initiation of ART in low- and middle- income settings - a systematic review. J Int AIDS Soc. 2014;17(1):19032. 10.7448/IAS.17.1.19032 PMC4122816 25095831

[17] Reed B, Hanson D, McNaghten A, Bertolli J, Teshale E, Gardner L, et al. HIV Testing Factors Associated with Delayed Entry. AIDS Patient Care STDS. 2009;23(9). 10.1089/apc.2008.0213 19694550

[18] Mimiaga MJ, Reisner SL, Grasso C, Crane HM, Safren SA, Kitahata MM (2013). Substance use among HIV-infected patients engaged in primary care in the United States: findings from the centers for AIDS research network of integrated clinical systems cohort. Am J Public Health.

[19] Owens DK, Davidson KW, Krist AH, Barry MJ, Cabana M, Caughey AB (2019). Screening for HIV infection: US preventive services task force recommendation statement. JAMA - J Am Med Assoc.

[20] Bradley KA, Debenedetti AF, Volk RJ, Williams EC, Frank D, Kivlahan DR (2007). AUDIT-C as a brief screen for alcohol misuse in primary care. Alcohol Clin Exp Res.

[21] Kitahata MM, Rodriguez B, Haubrich R, Boswell S, Mathews WC, Lederman MM (2008). Cohort profile: the centers for AIDS research network of integrated clinical systems. Int J Epidemiol.

[22] Spitzer, Robert L ; Kroenke, Kurt ; Williams JBW. Validation and Utility of a Self-report Version of PRIME-MD: The PHQ Primary Care Study. JAMA - J Am Med Assoc. 1999;282(18):1737–1744. 10.1001/jama.282.18.1737 10568646

[23] Bradley KA, McDonell MB, Bush K, Kivlahan DR, Diehr P, Fihn SD (1998). The AUDIT alcohol consumption questions: reliability, validity, and responsiveness to change in older male primary care patients. Alcohol Clin Exp Res.

[24] Ali R, Awwad E, Babor TF, Bradley F, Butau T, Farrell M (2002). The alcohol, smoking and substance involvement screening test (ASSIST): development, reliability and feasibility. Addiction..

[25] Crane HM, Lober W, Webster E (2007). Harrington, Robert D Crane PK, Davis TE, Kitahata MM. Routine collection of patient-reported outcomes in an HIV clinic setting: the first 100 patients. Curr HIV Res.

[26] Opravil M, Ledergerber B, Furrer H, Hirschel B, Imhof A, Gallant S, et al. Clinical efficacy of early initiation of HAART in patients with asymptomatic HIV infection and CD4 cell count. AIDS. 2002;16(January). 10.1097/00002030-200207050-00009 12131214

[27] Gras L, Kesselring AM, Griffin JT, Van Sighem AI, Fraser C, Ghani AC (2007). CD4 cell counts of 800 cells / mm 3 or greater after 7 years of highly active antiretroviral therapy are feasible. J Acquir Immune Defic Syndr.

[28] Rebeiro PF, Althoff KN, Lau B, Gill J, Abraham AG, Horberg MA (2015). Laboratory measures as proxies for primary care encounters: implications for quantifying clinical retention among HIV-infected adults in North America. Am J Epidemiol.

[29] Post FA, Wood R, Maartens G. CD4 and total lymphocyte counts as predictors of HIV disease progression. QJM An Int J Med. 1996;(Cdc):505–8. 10.1093/qjmed/89.7.505 8759490

[30] Tegger MK, Crane HM, Tapia KA, Uldall KK, Holte SE, Kitahata MM (2008). The effect of mental illness, substance use, and treatment for depression on the initiation of highly active antiretroviral therapy among HIV-infected individuals. AIDS Patient Care STDs.

[31] Finlayson TJ, Le B, Smith A, Bowles K, Cribbin M, Miles I, et al. HIV risk, prevention, and testing behaviors among men who have sex with men - national HIV behavioral surveillance system, 21 U.S. cities, United States, 2008. Morb Mortal Wkly Rep. 2011;60(SS-14):1–34. 22031280

[32] Stall R, Paul JP, Greenwood G, Pollack LM, Bein E, Crosby GM (2001). Alcohol use, drug use and alcohol-related problems among men who have sex with men: the urban men’s health study. Addiction..

[33] Wohl AR, Frye DM, Johnson DF (2008). Demographic characteristics and sexual behaviors associated with methamphetamine use among MSM and non-MSM diagnosed with AIDS in Los Angeles County. AIDS Behav.

[34] Zou G (2004). A modified Poisson regression approach to prospective studies with binary data. Am J Epidemiol.

[35] Greenland S (1987). Interpretation and choice of effect measures in epidemiologic analyses. Am J Epidemiol.

[36] SAMHSA. Key substance use and mental health indicators in the United States: Results from the 2018 National Survey on Drug Use and Health. HHS Publ No PEP19–5068, NSDUH Ser H-54. 2019;170:51–8.

[37] Spiller MW, Broz D, Wejnert C, Nerlander L, Paz-Bailey G (2015). Hiv infection and HIV-associated behaviors among persons who inject drugs — 20 cities, United States, 2012. Morb Mortal Wkly Rep.

[38] Irwin K (1996). L.; Valdiserri, Ronald O.; Holmberg SD. The acceptability of voluntary HIV antibody testing in the United States: a decade of lessons learned. AIDS..

[39] Rizza SA, MacGowan RJ, Purcell DW, Branson BM, Temesgen Z (2012). HIV screening in the health care setting: status, barriers, and potential solutions. Mayo Clin Proc [Internet].

[40] Lorenc T, Marrero-Guillamón I, Llewellyn A, Aggleton P, Cooper C, Lehmann A (2011). HIV testing among men who have sex with men (MSM): systematic review of qualitative evidence. Health Educ Res.

[41] Schulte MT, Hser YI (2014). Substance use and associated health conditions throughout the lifespan. Public Health Rev.

[42] Winkelman TNA, Admon LK, Jennings L, Shippee ND, Richardson CR, Bart G (2018). Evaluation of amphetamine-related hospitalizations and associated clinical outcomes and costs in the United States. JAMA Netw Open.

[43] Schwartz BG, Rezkalla S, Kloner RA (2010). Cardiovascular effects of cocaine. Circulation..

[44] Fazel S, Khosla V, Doll H, Geddes J (2008). The prevalence of mental disorders among the homeless in Western countries: systematic review and meta-regression analysis. PLoS Med.

